# Supplementary material for: Unveiling the Core Effector Proteins of Oil Palm Pathogen Ganoderma boninense via Pan-Secretome Analysis
Source: J Fungi (Basel). 2022 Jul 29;8(8):793. doi: 10.3390/jof8080793 (PMC9409662; doi:10.3390/jof8080793)
Supplement: Supplementary file 1 [file jof-08-00793-s001.zip › jof-1766095-Supplementary.pdf]

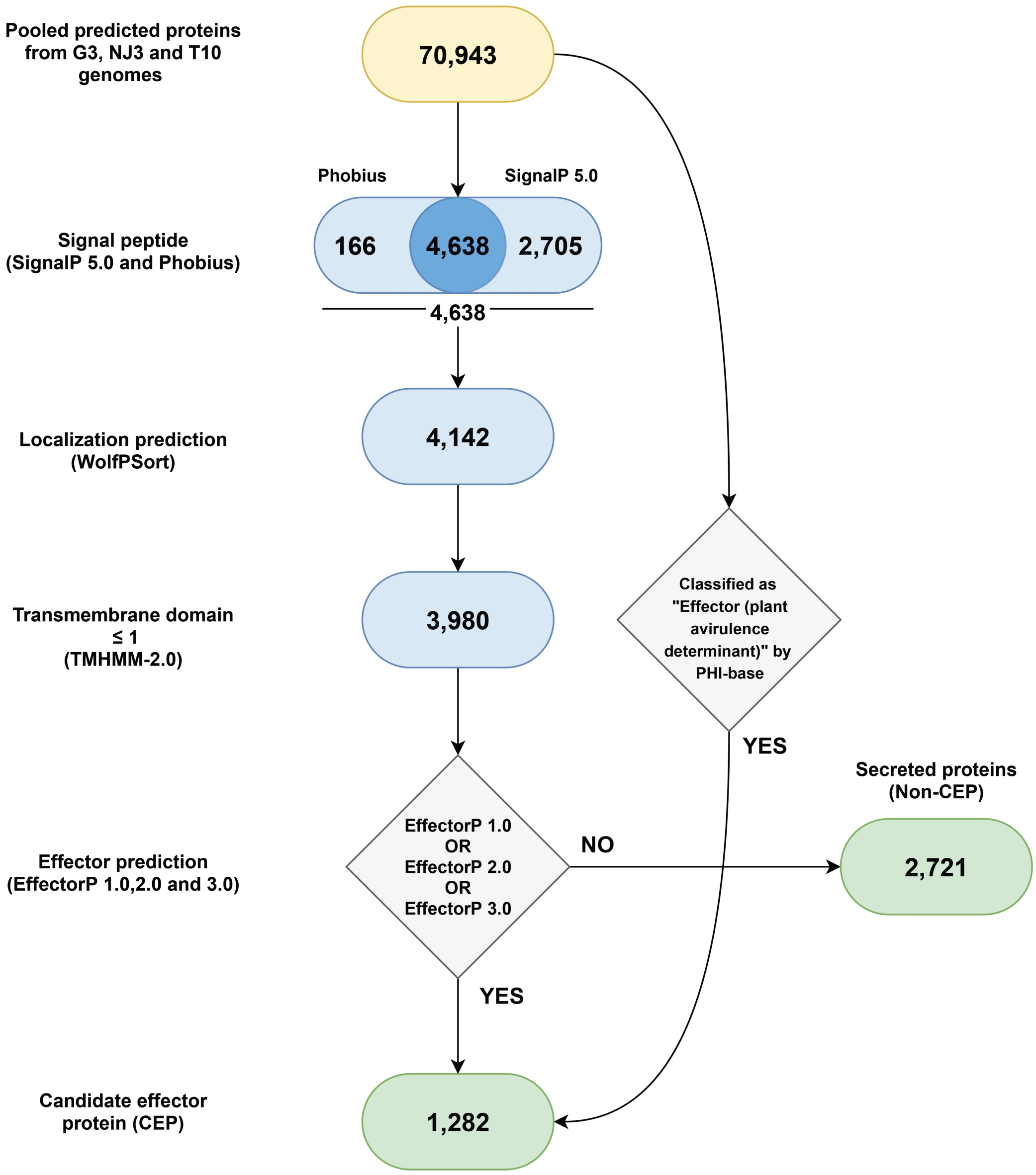

Supplementary Figure S1. Flowchart of the computational prediction pipeline for identifying secreted and effector proteins.

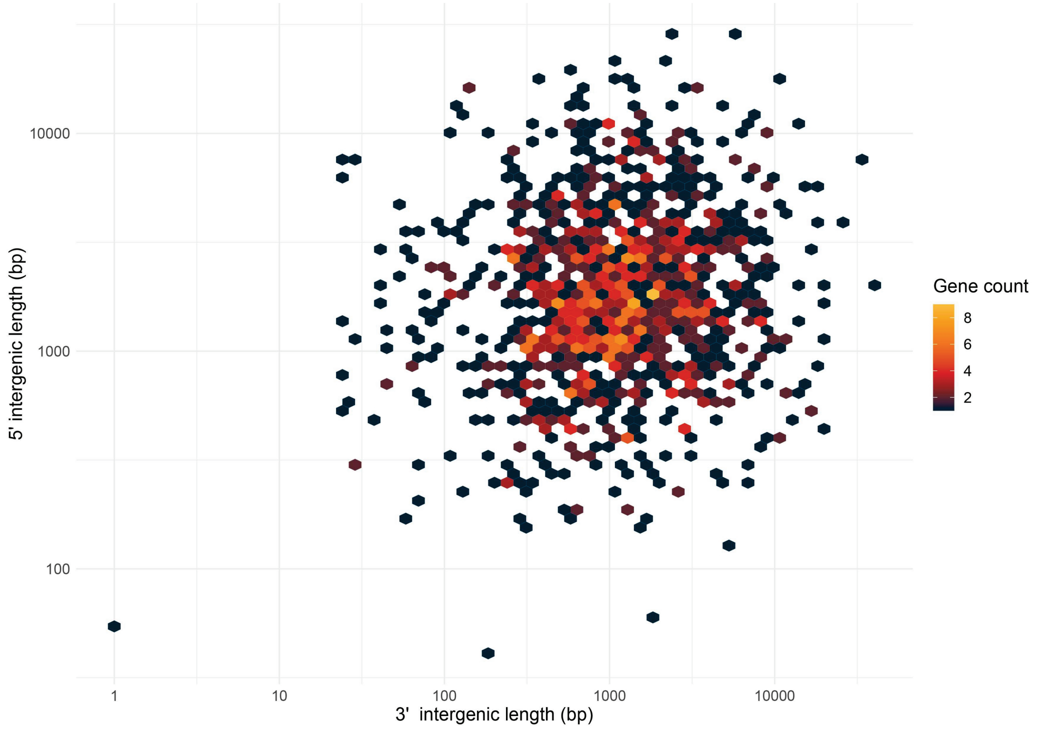

Supplementary Figure S2. Density plot illustrating the 5′ and 3′ intergenic distance for genes encoding secreted proteins.
